# Microbiome and Metabolome Insights into the Role of the Gastrointestinal–Brain Axis in Parkinson’s and Alzheimer’s Disease: Unveiling Potential Therapeutic Targets

**DOI:** 10.3390/metabo12121222

**Published:** 2022-12-05

**Authors:** Helena U. Zacharias, Christoph Kaleta, François Cossais, Eva Schaeffer, Henry Berndt, Lena Best, Thomas Dost, Svea Glüsing, Mathieu Groussin, Mathilde Poyet, Sebastian Heinzel, Corinna Bang, Leonard Siebert, Tobias Demetrowitsch, Frank Leypoldt, Rainer Adelung, Thorsten Bartsch, Anja Bosy-Westphal, Karin Schwarz, Daniela Berg

**Affiliations:** 1 Peter L. Reichertz Institute for Medical Informatics of TU Braunschweig and Hannover Medical School, 30625 Hannover, Germany; 2 Department of Internal Medicine I, University Medical Center Schleswig-Holstein, Campus Kiel, 24105 Kiel, Germany; 3 Institute of Clinical Molecular Biology, Kiel University and University Medical Center Schleswig-Holstein, Campus Kiel, 24105 Kiel, Germany; 4 Research Group Medical Systems Biology, Institute for Experimental Medicine, Kiel University, 24105 Kiel, Germany; 5 Kiel Nano, Surface and Interface Science—KiNSIS, Kiel University, 24118 Kiel, Germany; 6 Institute of Anatomy, Kiel University, 24118 Kiel, Germany; 7 Department of Neurology, Kiel University and University Medical Center Schleswig-Holstein, Campus Kiel, 24105 Kiel, Germany; 8 Research Group Comparative Immunobiology, Zoological Institute, Kiel University, 24118 Kiel, Germany; 9 Institute of Human Nutrition and Food Science, Food Technology, Kiel University, 24118 Kiel, Germany; 10 Department of Biological Engineering, Massachusetts Institute of Technology, Cambridge, MA 02139, USA; 11 Institute of Medical Informatics and Statistics, Kiel University and University Medical Center Schleswig-Holstein, Campus Kiel, 24105 Kiel, Germany; 12 Functional Nanomaterials, Department of Materials Science, Kiel University, 24143 Kiel, Germany; 13 Kiel Network of Analytical Spectroscopy and Mass Spectrometry, Kiel University, 24118 Kiel, Germany; 14 Neuroimmunology, Institute of Clinical Chemistry, University Medical Center Schleswig-Holstein, 24105 Kiel, Germany; 15 Institute of Human Nutrition and Food Science, Kiel University, 24107 Kiel, Germany

**Keywords:** gastrointestinal–brain axis, metabolomics, microbiome, neurodegenerative diseases, Parkinson’s disease, Alzheimer’s disease, therapeutic interventions, microbiome modelling

## Abstract

Neurodegenerative diseases such as Parkinson’s (PD) and Alzheimer’s disease (AD), the prevalence of which is rapidly rising due to an aging world population and westernization of lifestyles, are expected to put a strong socioeconomic burden on health systems worldwide. Clinical trials of therapies against PD and AD have only shown limited success so far. Therefore, research has extended its scope to a systems medicine point of view, with a particular focus on the gastrointestinal–brain axis as a potential main actor in disease development and progression. Microbiome and metabolome studies have already revealed important insights into disease mechanisms. Both the microbiome and metabolome can be easily manipulated by dietary and lifestyle interventions, and might thus offer novel, readily available therapeutic options to prevent the onset as well as the progression of PD and AD. This review summarizes our current knowledge on the interplay between microbiota, metabolites, and neurodegeneration along the gastrointestinal–brain axis. We further illustrate state-of-the art methods of microbiome and metabolome research as well as metabolic modeling that facilitate the identification of disease pathomechanisms. We conclude with therapeutic options to modulate microbiome composition to prevent or delay neurodegeneration and illustrate potential future research directions to fight PD and AD.

## 1. The Gastrointestinal–Brain Axis as a Potential Mediator of Microbiome Effects in Neurodegenerative Diseases

In the last few years, there has been a growing understanding of pathophysiological cascades and molecular changes involved in the manifestation of neurodegenerative diseases. This is particularly the case for the two most prevalent neurodegenerative diseases world-wide, i.e., Parkinson’s (PD) and Alzheimer’s disease (AD). However, triggering factors initiating these pathophysiological cascades, modulating factors influencing disease progression, as well as early interventional approaches addressing these factors remain elusive. Although monogenic forms of both PD and AD are known, the majority of cases are idiopathic with complex and heterogeneous etiological contributions including a multitude of possible genetic and/or environmental risk factors. Lifestyle factors such as physical activity and, in particular, diet, may very well constitute modifiable risk factors of PD and AD manifestation and progression [1,2]. Elucidating these factors is crucially important due to the enormous increase in PD and AD prevalence, which exceeds the increase that can be expected from an aging world population alone [3,4].

Based on pathological findings and the clinical observation of a slowly progressing neurodegenerative process, several phases of PD have been defined [5,6], which are divided into (1) a risk phase, i.e., a phase, in which genetic, environmental, and other factors contribute to the risk of PD, (2) a preclinical phase characterized by the initiation of progressive neurodegenerative pathology before any clinical symptoms or signs are evident, and (3) a prodromal phase defined by the emergence of observable signs or symptoms of neurodegeneration, including REM sleep behavior disorder (RBD), olfactory loss, autonomic dysfunction, depression (with or without comorbid anxiety), mild motor signs, and pathological imaging markers of the presynaptic dopaminergic system and the cardiac sympathetic system. This prodromal phase may precede the (4) clinical motor phase of PD, characterized by manifest bradykinesia with rest tremor and/or rigidity [7] for 10–20 years [8,9]. In all these PD phases, the onset and progression of motor and non-motor symptoms can differ tremendously between individuals [10]. Based on this heterogeneity, clinical and prodromal PD may be classified into subtypes with different pathomechanisms and patterns of spatial and temporal progression in the central nervous system (CNS) and peripheral nervous system (PNS) leading to diverse clinical manifestations [11]. For example, some clinical features such as early cognitive deficits, RBD, autonomic dysfunction, and some genetic risk factors are associated with faster progression [12]. Moreover, patients with RBD exhibit distinct patterns of α-synuclein pathology propagation and might indicate a body-first subtype rather than a brain-first subtype [13]. In addition, several biological processes inherent to normal ageing, environmental and life-style issues are relevant to the progression of PD [14,15].

In AD, an early deposition of amyloid and tau, as hallmarks of the neuropathological process in AD, has been shown in the precuneus and posterior cingulate followed by further accumulation in other cortical regions. This facilitates pathologic spread of tau from the medial temporal lobes to other cortical regions in AD, suggesting that this spatial–temporal gradient corresponds to disease progression and different clinical disease subtypes [16,17]. In AD, as well as “classical” AD, several disease subtypes with characteristic regional patterns of tau pathology have been classified that are characterized by differences in clinical phenotype, age, disease course, cognitive status, APOE genotype, and biomarker status. Furthermore, complementing four major subtypes based on the distribution of tau pathology and brain atrophy (typical, limbic predominant, hippocampal sparing, and minimal atrophy), several other clinical variants (non-amnestic, corticobasal, behavioral/dysexecutive, posterior cortical variants, etc.) have been delineated. These different subtypes and variants of AD are characterized by different patterns of key neuronal network dysfunction, in particular changes in the default-mode network. However, even in these subtypes, individual constellations of aforementioned pathologies, disease processes and their spatial and temporal relevance as well as risk/protective marker profiles may play a major role and are only partly understood [18,19,20,21,22,23].

The nervous system of the gastrointestinal (GI) tract, which contains 200–500 million neurons, is in close exchange with the CNS. This bidirectional communication is often referred to as the gut–brain axis. However, as it also involves the upper GI tract, including the mouth and its specific microbial environment, we hereafter use the broader term “GI–brain axis”. Several modes of communication along the GI-brain axis have been described, which can be summarized as neurochemical, endocrine, and immune interactions [24]. Yet, the breadth of mechanisms involved in this communication is only poorly understood. A growing body of research now suggests that our microbiota, the diverse and complex communities of commensal microbes that colonize all our body surface barriers, play a key role in the GI–brain axis, and may be involved in neurodegenerative diseases. Closely interconnected with the microbiome is the metabolome, the complete set of small molecules, called metabolites, which are intermediate or end-products of metabolism. Their involvement in neurodegenerative diseases currently also attracts wide interest in the research community.

In this review, we summarize the current knowledge on the association between microbial imbalance and neurodegeneration as exemplified for PD and AD. Furthermore, we review state-of-the-art association studies between neurodegeneration and the metabolome in PD and AD, the role of metabolic modeling in defining molecular pathways underlying those associations, as well as the potential of both the microbiome and the metabolome as novel therapeutic targets to treat neurodegeneration [25].

## 2. From the Microbiome to the Metabolome

Human microbiota are mostly composed of bacteria, but also contain archaea and microbial eukaryotes, along with their associated viral communities. The collection of genes encoded by these microbial communities defines the microbiome. Microorganisms produce diverse molecular compounds that directly influence host metabolism, prime immune responses, and shape physiology [26]. They also harbor complex surface markers that engage in direct contact interaction with host receptors or circulating proteins, which can either trigger anti- or pro-inflammatory responses [26]. As such, endogenous microbes are suspected to be strong contributors to our health via constant inter-organ interactions, also including the CNS [27]. However, detailed and mechanistic knowledge about these signaling pathways is limited, even though progress has been made recently [28]. As an example, an increasing body of evidence now points to a key role played by short-chain fatty acids (SCFAs) produced by gut bacteria, such as acetate, propionate, and butyrate, in long-range communications with the brain [28,29]. SCFAs are produced from the metabolism of indigestible materials such as complex fibers, contribute to preventing pathogen invasion, and participate in shaping the immune system [26]. Their downstream effects on host physiology are very diverse: while acetate is readily absorbed into the bloodstream and distributed to peripheral tissues, propionate is metabolized by the liver after absorption [30]. The majority of butyrate, on the other hand, is consumed locally by colonocytes as a primary fuel source [31]. The interplay between microbial metabolites and the CNS is further outlined in subsequent sections of this review.

### 2.1. Interrogating the Microbiome

Historically, culture-independent methods were used to characterize the diversity and structure of the microbiota by amplicon sequencing of phylogenetic marker genes (e.g., the 16S rRNA gene) [32]. Amplicon data are usually restricted in taxonomic, genomic, and functional information, limiting our understanding of the differences in microbial features that may exist between two given microbiomes. Today, deep shotgun metagenomic sequencing is used as the gold-standard approach to interrogate microbiomes [33,34]. Combined with sophisticated computational methods that reconstruct draft genomes [35], identify microbial lineages at the resolution of strains [36,37], or reconstruct gene repertoires [38] with detailed functional annotations [39], metagenomics provides high-dimensional and complex data that, when used alone or integrated with other -omics data, can reveal insightful associations between the microbiome and disease phenotypes [40,41].

Microbiome signatures of disease usually exhibit a loss of taxonomic diversity, decrease in the abundance of microbes that are suspected to be beneficial, and increase in abundance of potential pathobionts [42]. However, microbiome association studies have suffered from a lack of replicability across different cohorts [42] concerning the identification of microbiome features that are associated with disease. Both biological and methodological aspects contribute to replicability problems, and need to be taken into account in future GI–brain axis studies. In particular, recent studies showed that inter-individual variability is high in human microbiome data [43], and that geography can have a larger effect on microbiome variance than any other disease-relevant human trait, including drugs, diet, or genetics [44,45]. Therefore, large sample sizes, appropriate geographic (and/or lifestyle) representation and extensive surveys of metadata are needed to limit the effect of confounders and to draw reliable conclusions [40]. Importantly, technical protocols (e.g., to extract microbial DNA) and the choice of experimental or computational tools can explain more variance in microbiome sequencing results than single host traits [46,47]. Finally, the most recently developed statistical methods aim at accounting for the specific characteristics of microbiome data [42,48]. Microbiome data, which are count data, are compositional (quantification data for each taxa are usually in relative, and not absolute abundance), high-dimensional (hundreds or thousands of microbial taxa are detected among a given set of samples), sparse (a large fraction of microbial taxa are detectable only in a subset of samples), and overdispersed (variances of count data are larger than would be expected under a Poisson model) [48]. Accounting for these data characteristics helps improving association analyses and promoting cross-study comparisons. Overall, the microbiome research community needs to embrace experimental and computational standards that minimize batch effects, increase reproducibility, and promote cross-study meta-analyses [49].

For both PD and AD, differences in gut microbiome features compared to healthy controls have been observed [50,51]. It has been speculated that differences in the taxonomic diversity and composition of the GI tract microbiota results in perturbations of metabolic and immune–microbe interactions, thereby contributing to disease pathology. The causal role of a disturbed microbial homeostasis on pathogenesis can be supported by fecal microbiota transplant (FMT) experiments, in which disease phenotypes could be transferred from affected individuals to germ-free recipient animals through microbiome transfers alone. Thus, it has been postulated that dysbiotic changes of the microbiome are an important contributor to diseases such as inflammatory bowel disease (IBD) [52], PD [53], AD [54], and aging per se [55]. However, findings of FMT studies that used germ-free animals have been called into question due to the surprisingly high success rate of microbiome transfer experiments [56]. As such, mechanisms through which dysbiotic changes of the microbiome could contribute to disease processes in the host, and neurodegeneration in particular, are still poorly understood. A disbalanced microbiome could be characterized by an overabundance of pathogenic bacteria that are capable of releasing molecules, such as endotoxins, that may induce inflammation and compromise barrier integrity. Alternatively, perturbations in the quantity, or balance, of SCFAs are also suspected to be involved, as SCFAs have been shown to be key microbial mediators in the GI–brain axis [28].

### 2.2. Interrogating the Metabolome

**Figure 1:**
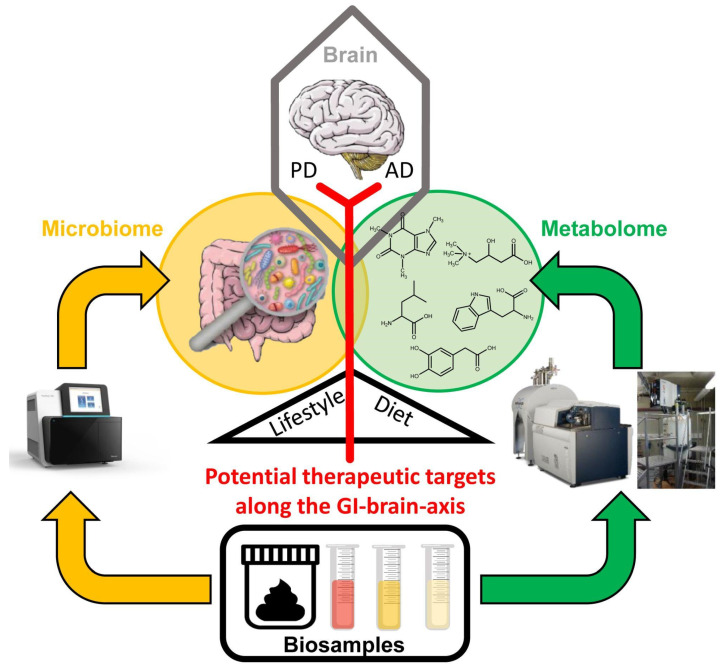
Schematic illustration of the gastrointestinal (GI)–brain axis (red) that could be modulated via potential therapeutic targets involving indirect (lifestyle/diet) and direct modulation of the microbiome (orange circle) and metabolome (green circle). Such therapeutic modulation of the GI-brain axis may represent a promising strategy for the early prevention of neurodegenerative processes in Parkinson’s and Alzheimer’s disease. Further research and analyses of biosamples regarding the microbiome and metabolome are needed and facilitated by methodological advances: sequencing allows the taxonomic and functional characterization of the microbiome (orange arrows) of stool samples of the gut and biospecimens from various other body sites. Metabolomics (green arrows) is facilitated by mass spectrometry (left) or nuclear magnetic resonance spectroscopy (right).

The comprehensive study of the metabolome in a particular biospecimen is the core goal of metabolomics (Figure 1) [57]. As the metabolome rapidly responds to both endo- and exogenous stimuli, metabolomics can provide a metabolic “snapshot” or “fingerprint” of the current state of an organism. It is thus able to offer new insights into the pathomechanisms underlying human diseases and identify potential therapeutic targets. Metabolomics studies can be conducted either in an untargeted or a targeted manner. Untargeted metabolomics tries to maximize the metabolome coverage of an investigated biospecimen, without any a priori metabolite selection. In contrast, targeted metabolomics measures a predefined set of metabolites, and often provides absolute quantification of their concentrations.

Metabolomics measurements in biospecimens are typically conducted by nuclear magnetic resonance (NMR) spectroscopy or mass spectrometry (MS) (Figure 1). NMR spectroscopy separates different metabolite signals according to their resonance frequencies within a magnetic field. MS, in contrast, identifies different metabolites by analyzing their mass-to-charge ratios. Considering the complex nature of biological samples, the majority of MS analysis methods involve prior analyte separation. Hyphenated techniques combine, e.g., liquid (LC) or gas chromatography (GC) with mass spectrometers. Compounds that are adequately volatile can be easily analyzed by GC–MS. In GC–MS, the electron impact ionization source allows neutral molecules to be ionized using an electron beam, and instantaneously fragments the entering molecules into a characteristic pattern [58,59,60]. LC–MS generally uses soft ionization techniques that mainly display the molecular ion species with only a few fragment ions. To overcome the resulting problem of rather poor information obtained from a single LC–MS run, tandem mass spectrometry (MS/MS) can be used. LC–MS/MS provides fragments through collision-induced dissociation of the molecular ions produced [61]. When compared to LC–MS based methods, GC–MS has advantages of a greater chromatographic resolution, a good retention of small compounds, and large spectral libraries [62,63]. However, the thermal stability of samples limits the metabolome coverage by GC-MS. Furthermore, several metabolites require derivatization, which might produce artifacts [63].

NMR spectroscopy requires very little sample preparation and measurements are highly reproducible both across time and across different lab facilities [64]. Likewise, NMR-based metabolomics is rather cheap, since only one internal standard is required to extract absolutely quantified metabolite concentrations from spectral data. This also facilitates absolute metabolite quantification in untargeted NMR metabolomics experiments. Furthermore, NMR experiments are non-destructive, and biospecimens can be re-used afterwards. NMR spectroscopy is, however, a rather insensitive analytical technique in comparison to MS, and acquired spectral data, especially from one-dimensional NMR experiments, are highly complex. Therefore, metabolite identification and absolute quantification are, to date, still very time-consuming processes, mainly conducted manually and not yet completely automatized. High spectral metabolite signal overlap can be partially compensated by two-dimensional NMR experiments, but at the expense of significantly increased measurement times. Nevertheless, these experiments can also provide further important structural metabolite information, which can improve the identification and characterization of unknown compounds.

MS-based approaches have the advantage of high sensitivity and selectivity, as well as high throughput and depth of coverage. The applicability of direct injection in metabolomics is extended by advanced instrumentation capable of high-resolution, accurate mass measurements, and tandem MS [65]. Fourier transform—ion cyclotron resonance mass spectrometers (FT-ICR-MS; Figure 1) are the most advanced mass analyzers in terms of information content and resolving power, with sub-parts-per-million mass accuracy [66] and the possibility of direct infusion mass spectrometry, which generates data in only a few minutes.

The most comprehensive coverage of the metabolome can only be achieved by a combination of different analytical techniques, e.g., NMR and different MS methods. Therefore, these techniques should be seen as complementary rather than as competing. For detailed information on conducting metabolomics experiments, we refer the interested reader to [57,67,68,69].

Most metabolome analyses to identify biomarkers for AD and PD are based on cerebrospinal fluid (CSF) [70] and blood specimens [71], including plasma [72] and serum [73]. As CSF has a more immediate connection to the brain than any other fluid, it directly reflects its metabolic changes [74]. The collection of blood samples is less invasive, acceptable for repeated measures, and most closely connected to CSF. Some studies have examined other biological matrices such as urine [73], feces [75], brain tissue [76], saliva [77], or sebum [78]. Urine is of great interest for biomarker identification, as it contains most of the body’s metabolic end products [79] and is therefore able to reflect comprehensive changes of metabolites in organisms [80]. In addition, urine represents a non-invasive biospecimen source. The fecal metabolome is also of particular interest since it more directly captures the complex interactions between the gut microbiome and the host [81].

## 3. Microbiome and Microbiome-Linked Metabolome Changes in Neurodegeneration

### 3.1. Microbiome Changes in Neurodegeneration

Accumulating evidence has underlined the putative involvement of the microbiota in neurodegenerative disorders in animal models. Alterations of intestinal microbial communities are observed in most PD mouse models [82]. Interestingly, microbiota from PD patients exacerbated neuroinflammation and motor dysfunction in germ free α-synuclein-overexpressing mice, emphasizing the potent detrimental effects of the microbiota in PD [83].

To date, most studies on PD-associated microbiota in humans are based on 16S rRNA-sequencing. Although many studies have identified alterations of fecal microbial diversity in PD, a high variability is observed in currently published datasets. Recently, several meta-analyses have attempted to provide a more unified view on microbial alterations occurring in PD [53,84]. The study by Plassais et al. indicated that microbial alpha-diversity is not significantly altered in patients with manifest PD or multiple sclerosis in comparison to healthy controls [84]. However, a second meta-analysis showed increased alpha-diversity in PD compared to controls, and suggested a link between disease and changes in the abundance of bacterial species, as well as intestinal inflammation [53]. These changes include an enrichment of the genera *Lactobacillus*, *Akkermansia*, and *Bifidobacterium*, as well as a reduction in the *Lachnospiraceae* family and *Faecalibacterium* genus, which both have been described as SCFA producers. These results were partly confirmed in a subsequent analysis indicating that increased *Akkermansia*, some species of which might possess mucolytic properties reducing gut wall integrity [85], and reduced *Roseburia* are consistently found in PD [86]. Importantly, similar microbial changes in the abundance of SCFA producing taxa have also been reported for prodromal stages and markers of PD [87,88]. In clinical PD, two longitudinal microbiome studies showed that PD patients with a more severe worsening of motor symptoms over time (~27 months and 12 months) had a lower abundance of SCFA producing bacteria (i.e., *Prevotella* and *Barnesiella*, respectively) compared to patients with stable or less severe progression of motor deficits [89,90]. Thus, for PD as a progressive neurodegenerative disease, the microbiome may play an early role in the prodromal as well as in more advanced clinical stages of the disease. However, the functional role(s) of specific microbial taxa for PD and/or AD remain to be further investigated as evidence on their specific GI–brain axis function(s) for the host and within complex microbial communities is still scarce.

Microbiota alterations in PD have also been shown using shotgun metagenomic sequencing, indicating that the frequency of a subset of bacterial genes may allow PD patients to be distinguished from healthy subjects, as well as from patients with multiple system atrophy (MSA) or AD [91]. Moreover, a functional metagenomic analysis suggested differences in the metabolism of SCFA precursors in PD compared to controls [92].

Noteworthy, most studies investigating a potential link between fecal microbiome and PD have suggested that microbiota-derived SCFA production may be altered in PD [93]. In particular, a reduction in fecal SCFA concentrations has been observed in PD patients [94], while the investigation of SCFA levels in serum or plasma have led to more conflicting results. A first study indicated that SCFA concentrations appear not to be significantly changed in the serum of PD patients [95], and an additional study indicated that serum SCFAs may help to distinguish between MSA and PD patients, but not between PD patients and control subjects [95,96]. On the contrary, more recent studies have reported increased plasma SCFA concentrations, particularly for acetate and propionate, in PD patients in comparison to control subjects [97]. Limited availability of SCFAs in the blood due to their fast metabolization along the GI tract may explain these discrepancies in part.

In AD, gut dysbiosis potentially triggers increased systemic inflammation, which in turn increases penetrability of the gut mucus barrier and leads to a more transmissible blood–brain–barrier. Thus, microbiome derived metabolites, such as lipopolysaccharides derived from bacterial cell walls (astrocyte activation), SCFAs (anti-neuroinflammatory), secondary bile acids (neurodegenerative), or tryptophan-related metabolites (neuroinflammatory), are more likely to reach the brain. A detailed review by Bairamian and colleagues [98] has covered the role of microbiota in AD more exhaustively. AD-associated dysbiosis comprises an increase of pro-inflammatory microbes and decrease in anti-inflammatory commensals. Bacteria of the Firmicutes phylum (including butyrate producers) are reduced in aged individuals [99] as well as in AD patients [100], and furthermore in the AD mouse models 5xFAD [101] and P301L [102]. Microglia are brain resident immune cells, which act neuroprotective in the homeostatic state (M0), but can act pro-inflammatory in a disease-associated microglia (DAM) state [74,98]. SCFA supplementation rescued an immature microglia phenotype in germ free mice [103]. Butyrate has been shown to alter microglia states towards the homeostatic M0 type [104]. Amyloid accumulation in aged humans with or without dementia was negatively correlated with butyrate and anti-inflammatory IL-10, whereas acetate, valerate, and proinflammatory cytokines have been positively correlated [105]. In the APP/PS1 mouse model, a fiber rich diet increased abundance of butyrate producing taxa, which led to reduced astrocyte activation and improved cognitive function, while propionate showed deleterious effects [106]. APOE, with the allele e4 being the biggest genetic risk factor for AD, is involved in the shift of microglia states from homeostatic M0 to DAM via expression of the neuroinflammation-associated TREM2 gene [107]. Interestingly, APOE4 carriers have been shown to have reduced levels of *Ruminococcaceae*, known butyrate producers, compared to the APOE2/E3 genotype [108], and loss of these bacteria was also observed in AD patients [100]. It is currently hypothesized that microbiome derived amyloid proteins (e.g., curli) could induce amyloid-beta or α-synuclein aggregation by acting as a seed [109]. Additionally, it has been shown that it is possible for α-synuclein to shuttle from gut to brain via the vagus nerve [110]. Microbial amyloid proteins might be able to take a similar route [51]. However, it is possible that for each, prodromal PD and prodromal AD, different subtypes may exist [11] that differ in routes of pathology and, possibly, the degree and/or nature of the contribution of the (gut) microbiome to the pathogenesis, increased risk of PD/AD and/or microbiome-dependent modification of disease progression. Thus, subtype differences in these regards may constitute an important aspect of heterogeneity between individuals in prodromal and clinical disease stages, and statistical findings of microbiome-phenotype associations might be stronger and more robust in specific subtypes.

### 3.2. Metabolomic Changes in Neurodegeneration

Several studies already investigated metabolome changes associated with PD (Table 1). Shao et al. identified several metabolites such as caffeine metabolites and fatty acids that were significantly decreased in plasma of PD patients compared to healthy controls [111]. Hatano et al. reported caffeine-related metabolites and purine derivatives as significantly decreased only during the initial stages of PD in the serum of PD patients [112]. Furthermore, increased levels of branched-chain amino acids (BCAAs) were found in patients with PD [113]. In urine, the levels of leucine and isoleucine were positively correlated with disease stage in idiopathic PD patients [113]. In line with this finding, milk consumption (but not fermented milk intake) was associated with increased risk of PD [114,115]. Other authors however found a negative correlation between plasma BCAAs or essential amino acids (EAA) and Parkinson’s disease scores [116]. In these patients, whey protein supplementation increased plasma BCAAs and EAAs and led to an increase in plasma reduced glutathione and a reduction in homocysteine levels at unchanged levels of motor deficits as indicated by clinical ratings. Furthermore, a dysregulation of metabolites associated with carnitine metabolism was observed in sebum [78] and plasma [117]. Carnitine-dependent oxidation of fatty acids is an alternative way of energy production in mitochondria. Therefore, a disturbance of the carnitine metabolic pathway could be related to the mitochondrial dysfunction observed in PD [117]. Significantly lower plasma or serum levels of tryptophan and kynurenine were reported for PD patients, indicating an involvement of this particular pathway in PD pathogenesis [72]. In a rotenone-induced rat model of PD, dietary tryptophan supplementation was shown to protect against rotenone-induced neurotoxicity to ameliorate motor deficits, which may be mediated through activating the aromatic hydrocarbon receptor pathway [118]. Similarly, metabolic profiling of whole blood samples showed increased levels of leucine in de novo PD patients compared to controls as well as higher levels of tryptophan metabolites, including kynurenine and xanthurenic acid, in PD patients compared to controls [71].

Recent studies on metabolomics in AD are summarized in Table 1. Similar to PD, alterations in serum acylcarnitine composition have been reported in incident AD and associated with cognitive decline [119]. An analysis of feces specimens revealed higher ammonia and lactic acid concentrations in subjects with dementia [120]. Targeted metabolomics analyses in serum and brain tissue demonstrated alterations of bile acid metabolism in AD, resulting in a higher proportion of secondary bile acids in comparison to healthy subjects [121,122,123]. Bile acids are considered important endocrine and paracrine effectors, directly linking liver homeostasis and intestinal co-metabolism with the CNS. Multiple studies investigated how bile acids cross the blood–brain–barrier and how they are involved in signaling circuits, emphasizing the role of the GI–(liver)–brain axis in AD [124,125,126]. Intestinal abundance of the genus *Faecalibacterium* correlated negatively with disease severity in dementia, which was confirmed in further studies in AD and PD [93,127,128]. The role of *Faecalibacterium* as an important butyrate fermenter with anti-inflammatory effects has already been discussed for IBD [129]. A multivariable, blood-based metabolite panel might be promising to differentiate AD patients from controls and other types of dementias [130].

**Table 1:** Recent metabolomics studies in (a) Parkinson’s and (b) Alzheimer’s disease.

Publication	Study Question	Analytical Method	Sample Matrix	Additional Measurements	Study Population	Findings
**(a)** **Parkinson’s disease**						
[97]	compare fecal and plasma levels of different SCFA subtypes in patients with PD and healthy controls	GC-MS and LC-MS/MS	feces and plasma	total fecal DNA	96 PD patients and 85 controls	reduced fecal SCFAs and increased plasma SCFAs observed in patients with PD and correlated to the abundance of pro-inflammatory *Clostridiales* and *Ruminococcus* species and clinical severity of PD
[131]	characterize metabolite and lipoprotein profiles of newly diagnosed de novo drug-naïve PD patients	NMR	serum	-	329 subjects including de novo drug-naïve PD patients, PD patients with advanced disease status, and healthy controls	metabolic differences between newly diagnosed de novo drug-naïve PD patients and healthy controls, which were more pronounced in male patients (particularly acetone and cholesterol); metabolic differences between de novo drug-naïve PD patients and advanced PD patients; metabolic differences between advanced PD patients and healthy controls
[132]	clinical relevance of microbiome and metabolome alterations in PD	NMR and LC-MS	feces	16S-sequencing of fecal microbiota	104 PD patients, 96 control subjects	increased abundance of *Bacteroides fragilis*, *Lactobacillus acidophilus*, unclassified *Megasphaera* and unclassified *Gammaproteobacteria*; greatest effect size for NMR-based metabolome; SCFAs, lipids, TMAO, ubiquinone and salicylate concentrations vary in PD patients; low SCFA levels correlate with poorer cognition and low BMI; low butyrate levels correlate with worse postural instability-gait disorder scores
[133]	Integration of longitudinal metabolomics data with constraint-based modeling of gut microbial communities	LC-MS	EDTA plasma	16S-sequencing of fecal microbiota	30 PD patients, 30 control subjects	combined omics-methods suggest correlation between sulfur co-metabolism and PD severity; dopaminergic medication affects lipidome; levels of taurine conjugated bile acids correlate with severity of motor symptoms; *A. muciniphila* and *B. wadsworthia* are predicted to alter sulfur metabolism
[134]	alterations in gut microbiota might be accompanied by altered concentrations of amino acids, leading to PD	LC-MS, GC-MS	feces	16S-sequencing of fecal microbiota	PD patients and healthy controls	greater abundance of *Alistipes*, *Rikenellaceae_RC9_gut_group*, *Bifidobacterium*, *Parabacteroides*, while *Faecalibacterium* was decreased in PD feces specimens; fecal BCAAs and aromatic amino acids concentrations were significantly reduced in PD patients compared to controls
[135]	finding a cause-effect relationship between intestinal dysbiosis and PD	GC-MS	feces	16S-sequencing of fecal microbiota	64 PD patients, 51 control subjects	alteration of fecal metabolome regarding lipids, amino acids, vitamins, cadaverine, ethanolamine and hydroxy propionic acid; severe metabolomic alterations correlate with abundance of bacteria from the *Lachnospiraceae* family
[136]	identification of early biomarkers for PD	FT-ICR-MS	CSF	-	31 patients, 95 control subjects	243 metabolites were found to be affected in PD; 15 metabolites are predicted to be the main biological contributors; network analysis showed connection to Krebs-Cycle, possibly displaying mitochondrial dysfunction
[137]	Integrative metabolic modeling to identify roles of gut microbiota in host metabolism contributing to PD pathophysiology	LC-MS	serum	-	31 early-stage L-DOPA-naïve PD male individuals, 28 matched controls	functional analysis reveals increased microbial capability to degrade mucin and host glycans in PD; personalized community-level metabolic modeling reveals microbial contribution to folate deficiency and hyperhomocysteinemia observed in patients with PD; decreased capacity to produce SCFAs by *Bacteroides* and *Prevotella* species observed
[111]	untargeted metabolomics approach to investigate metabolic changes associated with PD	LC-MS	plasma	-	223 PD, 169 healthy controls, 68 neurological disease controls	significant reductions in fatty acids and caffeine metabolites, elevation of bile acids; metabolite PD panel with 4 biomarker candidates: FFA10:0, FFA12:0, indolelactic acid and phenylacetyl-glutamine
[78]	investigating sebum as potential diagnostic tool for PD; identify PD biomarkers in sebum	LC-MS	sebum	-	80 drug-naïve PD, 138 medicated PD, 56 healthy controls	10 metabolites present in samples of drug-naïve and treated PD patients associated with carnitine pathway and sphingolipid metabolism pathway
[71]	compare metabolomic profiles of whole blood obtained from treated PD patients, de-novo PD patients and controls, and study perturbations correlated with disease duration, disease stage and motor impairment	GC-MS	blood	-	16 de-novo PD, 84 treated PD, 42 healthy controls	most prominent differences in butanoic acid and glutamic acid
[138]	identify distinct volatiles-associated signature of PD	GC-MS	sebum	-	43 PD, 21 healthy controls	altered levels of perillic aldehyde, hippuric acid, eicosane, and octadecanal in PD specimens
[117]	characterization of metabolic patterns in PD plasma specimens	LC-MS	plasma	-	28 PD, 18 healthy controls	17 significantly altered metabolites associated with glycerol phospholipid metabolism, carnitine metabolism, bile acid biosynthesis and tyrosine biosynthesis
[72]	identify candidate metabolic biomarker(s) and pathomechanistic pathway(s) of PD	LC-MS	plasma	-	discovery cohort including 82 PD, 82 healthy controls; validation cohort including 118 PD, 22 Huntington’s Disease, 47 healthy controls	dopamine and putrescine/ornithine ratio upregulated in PD, octadecadienylcarnitine C18:2, asymmetric dimethylarginine, tryptophan, and kynurenine downregulated in PD
[113]	urinary metabolic profiling of idiopathic PD patients at three stages and normal control subjects	GC-MS, LC-MS	urine	-	92 PD, 65 healthy controls	18 differential metabolites associated with BCAA metabolism and steroid hormone biosynthesis
[93]	identify associations between intestinal microbiome, intestinal digestive function, and influence of systemic microbial metabolites on PD	LC-MS	feces, serum	-	197 PD, 103 healthy controls	different intestinal microbiome composition in PD patients, with increased abundance of *Akkermansia* and *Bifidobacterium* and decreased abundance of *Faecalibacterium* and *Lachnospiraceae*; intestinal microbiome in PD patients had reduced capacity of carbohydrate fermentation and butyrate synthesis and showed increased proteolytic fermentation
**(b)** **Alzheimer’s disease**						
[121]	investigate role of bile acid composition in AD	LC-MS	serum	-	1464 subjects (370 cognitively normal, 284 early MCI, 505 late MCI, 305 AD patients)	in AD, cholic acid levels as a primary bile acid are significantly decreased and levels of the secondary bile acid deoxycholic acid are increased; levels of deoxycholic acid conjugated with taurine and glycine are also increased
[127]	investigating the metabolic output of gut microbiome dysbiosis in AD	LC-MS	feces	16S-sequencing of fecal microbiota	21 patients, 44 control subjects	in AD, 15 gut bacterial genera appear to be altered, 7 of those genera are associated with different series of metabolites; combination of bacterial genera *Faecalibacterium* and *Pseudomonas*, combined with 4 metabolites was able to discriminate between AD patients and controls
[120]	identify the relationship between microbiome-associated metabolites and dementia	LC, ion chromatography, GC-MS	feces	classification of fecal bacteria by T-RFLP	82 control subjects, 25 patients	fecal ammonium and lactic acid were identified as markers for dementia
[128]	identify key microbial taxa that participate in the gut-brain axis	CE-FTMS	mouse brain	16S-sequencing of fecal microbiota	21 control subjects, 15 patients with MCI, 7 AD patients	*Faecalibacterium prausnitzii* was identified to participate in the gut-brain axis as its abundance decreased in patients with MCI, correlating with cognitive scores; oral treatment of GMO mice with Aβ-induced cognitive impairment with *F. prausnitzii* improved cognitive impairment and altered metabolic profile in brain tissue specimens
[123]	connecting bile acid profiles with standard biomarkers of AD progression	LC-MS	serum	imaging of brain atrophy with MRI, assessment of β-amyloid and tau deposits with PET	305 control subjects, 98 subjective memory complaint patients, 284 early MCI patients, 505 late MCI patients, 305 AD patients	different bile acid profiles associated with Aβ1-42 in CSF and with p-Tau181 in CSF
[122]	metabolomic profiling of bile acids in serum and brain of AD patients	LC-MS	serum and brain tissue	metabolomic analysis of serum and brain samples were also performed in mice	10 AD patients, 10 healthy subjects	serum levels of cholic acid in AD patients decreased; concentration of taurocholic acid reduced in brain tissue
[119]	identification of novel biomarkers for improved risk prediction in AD	LC-MS	serum and brain tissue (post mortem)	follow-up analysis of serum metabolome after 4.5 years	serum: 97 patients with MCI, 433 healthy subjects; brain (post mortem): 28 AD patients, 32 patients with MCI, 52 healthy subjects	peripheral and systemic metabolome appears to have minor overlaps; three serum acetylcarnitines identified as negative predictors for incident AD and cognitive decline; another 13 metabolites were found as predictors for longitudinal change in cognition

## 4. Metabolic Modeling of the Gut–Brain-Axis

### 4.1. Constraint-Based Metabolic Modelling

**Figure 2:**
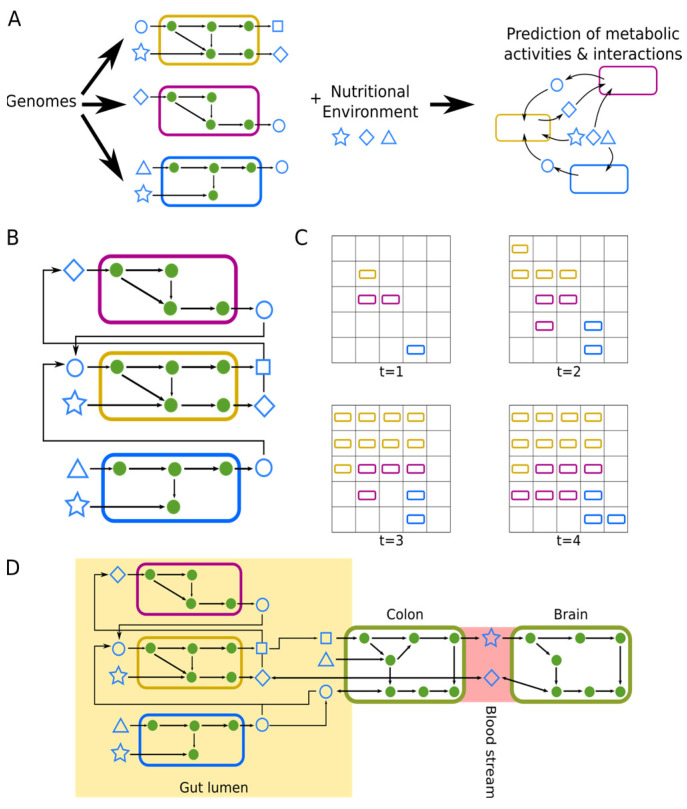
Microbial community modeling approaches. Circles correspond to metabolites, arrows to reactions. Shapes indicate exchanged metabolites. (**A**) General modeling approach. Genomes of bacterial species are translated into their corresponding metabolic network models. Additionally, information on the nutritional environment of the community is added (e.g., reported dietary uptake of a study participant). Subsequently, metabolic activities in individual bacterial species and metabolic exchanges between them can be predicted. (**B**) Community flux balance analysis. Bacterial metabolic networks are combined into a community level metabolic network and it is assumed that bacteria optimize their respective metabolic networks for most efficient community growth. (**C**) Individual-based modeling of microbial communities. Individual bacterial metabolic networks are simulated in a grid-like environment over time. Metabolic interactions occur as part of the secretion/consumption of metabolites by individual bacteria and diffusion of metabolites between grid cells. (**D**) Whole-body modeling. Metabolic networks of individual bacteria are joined with metabolic networks representing individual host tissues. Metabolic exchanges between bacteria and colon occur via a luminal compartment, metabolic exchanges between host tissues are mediated by the blood stream.

A major challenge for microbiome-based approaches especially in neurodegeneration is to deduce molecular mechanisms through which the microbiome could drive disease processes from associations between microbiome composition and disease phenotypes. This is due to the immense complexity of different microbiomes often comprising hundreds to thousands of species with a genetic markup about 150 times larger than that of the host [139,140]. It is further complicated by the large number of factors influencing microbiome composition, which makes it often impossible to distinguish whether changes in microbiome composition are caused by a disease or are causally involved in its pathogenesis [141,142]. One way to alleviate this problem is the utilization of mechanistic modeling approaches that allow to translate changes in microbiome composition to the potential change in the underlying molecular function of the microbiome [143,144]. One particularly important approach in this regard is constraint-based metabolic modeling that represents individual bacterial taxa as well as the host by their respective metabolic networks [143,144]. Taking into account the nutritional environment of the microbial community, these approaches then use the metabolic networks of the individual species together with an optimization approach to predict metabolic activities in individual species, metabolic exchanges between species, and metabolic exchanges with the host (Figure 2A). Importantly, these approaches can incorporate compositional information and other types of molecular data such as transcriptomic, proteomic, or metabolomic data, to provide hypotheses about the functional consequences of observed differences in microbiome composition [145,146,147].

Major approaches that are employed in this context are community flux balance analysis [148], individual-based modeling of microbiome metabolism [149], and whole-body modeling [150]. Community flux balance analysis combines the metabolic networks of individual species into a common compartment and optimizes the total amount of bacterial biomass produced by the entire community [148] (Figure 2B). This method assumes that bacterial species are using their metabolic networks such that the entire community produces the highest amount of bacterial biomass possible and hence assumes some intrinsic cooperation in the organization of metabolic fluxes between species. In contrast, individual-based modeling approaches such as BacArena that are also able to account for temporal dynamics, optimize the metabolic networks of bacterial species individually [149] (Figure 2C). As a consequence, metabolic interactions in BacArena mostly arise from one bacterial species excreting a product that it does not metabolize further which is taken up by another species that has that capability. In contrast, whole-body modeling aims to build integrated metabolic networks of the host and the metabolic networks of microbial species [150] (Figure 2D). These models allow tracing metabolic pathways connecting host and microbiota and thereby are able to propose molecular metabolic pathways through which the microbiome could influence disease processes in the host. Hence, whole-body modeling is able to explicitly model also metabolic interactions along the gastrointestinal–brain axis.

An additional important feature of these modeling approaches is that they enable the prediction of the outcome of perturbations. Hence, they are able to predict specific interventions such as supplementation of nutrients or probiotics that counteract disease-associated microbiome functions and therefore could be an essential component in the rational design of microbiome-based therapies counteracting neurodegeneration.

### 4.2. Microbial Community Modeling Yields Insights into Neurodegenerative Disease-Associated Changes in Microbiome Metabolic Activity

Constraint-based microbial community modeling approaches have already seen an application in a large number of different disease contexts, particularly IBD [151,152], type 2 diabetes [153], and PD [137,154]. In the context of IBD, community flux balance analysis was used to assess disease-associated changes in predicted metabolic activities of the microbiome and propose specific metabolic interventions that could counteract these changes [151]. In this study, particular changes in microbial sulfur metabolism were observed which is well in line with metabolomic observations [155]. Moreover, in another study, profound differences in ecological interactions within the microbiome that were predictive of anti-inflammatory therapy success were observed [152]. An interesting link to potential microbiome-based therapeutic approaches was drawn in a recent study investigating the contribution of the microbiome to the therapeutic effect of the anti-diabetic drug metformin [153]. It was found that in the roundworm *Caenorhabditis elegans*, the effect of metformin was mediated by bacterial production of the potential neurotransmitter agmatine. Using microbial community modeling on the gut microbiome of type 2 diabetic patients demonstrated also an increased capacity to produce agmatine in humans taking metformin. Interestingly, agmatine has previously been shown to have a neuroprotective effect in PD [156] as well as in AD [157]. Microbial production of agmatine upon metformin exposure could hence contribute to observed beneficial effects of metformin in AD [158] and PD [159]. In the context of PD, microbial community modeling was used to assess changes in gut microbiome metabolic capacity tied to disease severity [159]. It was found that sulfur-containing compounds such as cysteine-glycine and methionine showed an association with PD. Similarly, another study identified differences in metabolic capacities of individual microbial species that showed an association with PD [137], which were reflected in serum metabolomics data. Interestingly, in the same study, changes in microbial sulfur metabolism were observed.

## 5. The Microbiome as Therapeutic Target in Neurodegenerative Diseases

The emerging role of the microbiome as a potential driver of neurodegenerative diseases opens up new possibilities for targeted, causal therapies. In this context, two fundamentally different approaches to target the microbiome emerge. The first approach are changes in lifestyle that would largely constitute a therapeutic strategy without relevant adverse side-effects or safety concerns, but with high demands regarding personal initiative and adherence. The second therapeutic strategy entails the direct modulation of microbiome composition either through targeted modulation of the abundance of microbial species of interest or a complete replacement of the microbiome through fecal transplants.

### 5.1. Changes in Lifestyle: Diet and Exercise

As discussed, diet is one of the strongest factors influencing microbiome composition [160]. For neurodegenerative diseases, a variety of animal and observational studies have indicated beneficial effects of different forms of diet and nutritional habits. Of particular interest is the Mediterranean diet, which is associated with a decreased risk for PD and AD [161]. Parts of the neuroprotective or anti-inflammatory effects of the Mediterranean diet could be mediated by the microbiome [162]. One important aspect of this diet is the increased intake of fibers, which forms a direct link to microbially produced SCFAs [30]. Apart from effects on the intestinal and endocrinological system, SCFAs have been associated with positive effects on immunologic functions, including modulation of microglia and T-cell function in the ENS and CNS [163]. However, their specific role in neurodegenerative diseases remains somewhat convoluted [25]. Another important constituent of the Mediterranean diet are secondary plant compounds such as polyphenoles. Around 90–95% of total polyphenol intake may accumulate in the large intestine, where they become available for fermentation by the gut microbiota. Polyphenols and their degradation products (e.g., hydroxybenzoic acids) have been reported to inhibit the formation of α-synuclein misfolded aggregates, reduce mitochondrial dysfunction-induced oxidative stress, and inflammatory responses [164,165]. In contrast, the Western diet, including highly-processed, high-fat, and high-sugar foods, has been associated with pro-inflammatory properties, which have been linked to AD pathology and an increased risk for PD [166], with parts of these effects seemingly mediated by the microbiome [167]. The ketogenic diet has also been considered for its potential health benefits in neurological diseases, including PD, for which rodent models indicate that effects might be mediated by the gut microbiome [168].

Results from cohort studies and meta-analyses that focus on single food items such as dairy products and alcohol or single nutrients such as calcium, antioxidants, B-vitamins, and n-6 or n-3 polyunsaturated fatty acids have shown inconsistent results (for a review see [169]). Since diet is a multidimensional exposure of components with different health effects, a diet intervention based on an individual’s dietary patterns might have more favorable effects if it alters intake of multiple foods that may lead to a combination of many smaller effect sizes [170]. On the other hand, health effects of dietary patterns may depend on genetic risk alleles such as the APOE4 genotype as well as on the gut microbiome. Compared to vegans, omnivores produce significantly higher levels of the atherosclerosis-promoting trimethylamine-N-oxide (TMAO) after eating a protein-rich meal because several bacterial taxa that form the TMAO precursor trimethylamine have been reported to be more abundant in omnivores than in vegans [171]. The Nutrition for Dementia Prevention Working Group recently proposed a roadmap for future studies in nutrition and dementia prevention [170]. According to their recommendations, diets should be designed based on multiple neuroprotective dietary or nutrient components that can be applied in interventional trials. In addition, smaller personalized trials should be performed that consider genetics, omics, microbiome, and nutrient exposures and are guided by biomarkers that reflect brain functions.

Another important aspect of lifestyle as a therapeutic option for targeting the microbiome is exercise, which was shown to decrease the risk for neurodegeneration, induce neurorestorative and neuroprotective effects, and modulate disease progression in animal and human observational studies [172,173]. In this respect, a variety of rodent model studies revealed alterations of the gut microbiome following different forms of exercise, in interaction with, but also independently from dietary changes [174,175]. Similar effects have been observed in human studies, with alterations of microbial diversity observed in athletes and following different forms of exercise [176]. However, clinical interventional studies linking exercise-induced changes in the microbiome and direct health benefits in neurodegenerative diseases are still missing. Additionally, especially for PD, the interaction between exercise effects on gut motility and changes in the intestinal flora should be further elucidated.

Taken together, evidence from human studies confirming a direct link between lifestyle changes, microbiome alterations, and clinical benefits in neurodegenerative diseases are still needed. Moreover, while the potential therapeutic role of SCFAs needs to be clarified, available studies strongly support positive effects of a high-fiber, Mediterranean diet, and regular exercise, which should be mechanistically further elucidated to specifically advise them as low-risk therapeutic options for neurodegenerative diseases.

### 5.2. Prebiotics and Probiotics

Following accumulating evidence of a prominent role of the GI-brain axis in neurodegenerative diseases, researchers and patients placed high hopes in the use of prebiotics (nutrients supporting beneficial microbial strains), probiotics (beneficial strains) or combinations thereof referred to as synbiotics, to target the microbiome. In this respect the use of prebiotics has a high overlap to positively rated foods from dietary studies. Important prebiotics include fructooligosaccharides (FOS), galactooligosaccharides (GOS), polyunsaturated fatty acids (PUFA), and plant polyphenols. For neurodegenerative diseases, especially the neuroprotective, anti-inflammatory, and antioxidative effects of polyphenols and PUFAs have been described [164,177]. So far, a variety of animal and human studies have examined behavioral and neuropsychiatric effects of prebiotics, including effects on anxiety, depression, and memory function [178,179]. In AD mouse models, prebiotics such as plant polyphenols and oligosaccharides have been linked to an improvement of cognitive function and modulation of amyloid or tau pathology, in association with microbiome diversity and metabolism [180]. In contrast, very limited studies have been performed for PD, examining mainly the role of prebiotics (and probiotics) on constipation in clinical PD [181]. A first interventional, monocentric, open-label clinical trial RESISTA-PD (NCT02784145) that aimed at altering fecal SCFAs by an 8-week prebiotic intervention with resistant starch (RS) could demonstrate that in PD patients treated with RS, fecal butyrate concentrations increased significantly and fecal calprotectin concentrations dropped significantly after 8 weeks of RS therapy and that this prebiotic approach is safe and well-tolerated in PD [182]. Larger, blinded studies evaluating clinical outcome parameters are needed to substantiate this observation. Concerning probiotics, various animal and few human studies have shown behavioral and neuropsychiatric effects including effects on cognitive function or fatigue [183,184]. Accordingly, first interventional studies in AD and PD have been conducted, showing potential clinical benefits of microbiome alterations [185,186]. In summary, despite great interest in the use of prebiotics and probiotics to target microbiome-associated disease progression in neurodegeneration and first promising results, evidence from clinical studies is not sufficient, yet, for an official medical recommendation to use probiotics or prebiotics in AD or PD.

### 5.3. Antibiotics

The other route besides proliferation promotion with pre- and probiotics is the suppression of invasive or overabundant species through antibiotics. The treatment with broad-spectrum antibiotics is considered to have severe microbiome-related side effects such as microbiome dysbiosis [187]. For example, such a treatment has decreased the survival rates of patients with cancer [188] indicating the role of homeostasis for the overall health of a patient. In addition, intake of antibiotic medication has been suggested to increase PD risk in healthy individuals [189]. Therefore, caution is warranted with more general antibiotic treatments, while more targeted antibiotic interventions might be more promising. For instance, the overabundance of specific species can trigger the release of inflammatory mediators in AD patients, e.g., *Helicobacter pylori* [190]. Even viral infections such as herpes simplex virus (HSV) type 1 have been identified as possible risk factors in AD [191]. Antibiotic targeting of individual species inside the microbiome could cut feedback loops and synergistic effects important for the modulation of the overarching disease. For chronic peptic ulcers, this has already been implemented for a long time by specifically targeting *H. pylori* [192]. Another example is the treatment of *Clostridioides difficile* infections (CDI), where the switch from a broad-spectrum antibiotic to a specifically targeting, microbiome-sparing antibiotic could reduce CDI recurrence levels by 60% compared to the standard clinical therapy [193].

Still, in addition to interfering with the biochemical pathways of the pathogens, solid-state structural mechanisms could be proposed as well. Specifically antibiotic, but biocompatible particles such as zinc oxide micro-tetrapods [194] have already been employed to facilitate an immune response against HSV [195,196,197,198]. In this case, instead of a classic pharmaceutical effect through the release of zinc ions, the structural effect of binding virion glycoprotein groups to designed surface oxygen vacancies is used. For HSV, the experimental evidence elucidated the mechanism: a capturing of the nanoscopic virion to the microscopic tetrapodal zinc oxide surface was observed. Thus, the tetrapods acted as a virostatic platform. From there, antigen-presenting cells identified the immobilized viruses and thus triggered the immune system via the CD4/CD8 signaling pathway against herpes simplex viruses in a mouse model. As herpes simplex is one risk factor for AD, such a therapy could also reduce the overall AD risk.

This solid-state structural strategy is also applicable for bacteria. Structural differences cause differences in the antibacterial efficacy [195,196,197]. By using specific binding elements on top of the micro crystals, these can be tailored even more for a microbiome-sparing antibiotic targeting. By chemically altering the surface structure of the tetrapodal zinc oxide particles, the binding specificity towards other proteins for simultaneously anti- and prebiotic purposes could be achieved [195,196,197]. Such a combined approach may significantly impact the species in the microbiome as well as break negative and enhance positive feedback towards homeostasis.

### 5.4. Fecal Transplants

The possibility to alter the microbiome via fecal transplants (fecal microbiota transplantation, FMT) from healthy individuals has gained attention as a potential therapeutic option in neurodegenerative diseases. Fecal transplants are an evidence-based and recommended treatment for recurrent CDI [199,200]. Additionally, FMT has been increasingly discussed as a therapeutic option for other gastrointestinal disorders, particularly IBD and constipation [201,202,203]. Moreover, mouse models have revealed effects of FMT on neurobehavior [204,205] and on immunological pathways [205,206]. Regarding neurodegenerative diseases, several mouse model studies investigated effects of FMT on dysbiosis, protein accumulation, inflammation and clinical impairment. In PD, FMT treatments in the rotenone rodent model restored gut dysbiosis, inhibited neuroinflammation and improved gastrointestinal and motor dysfunction [207,208]. Similar results for the modulation of gut microbiome and neuroinflammation have been seen for the AD mouse model, in which FMT additionally reduced amyloid-ß and tau pathology, accompanied by improvements of cognitive function [209,210]. However, so far only few case reports and small case series have observed positive effects of FMT in patients with PD, demonstrating primarily an improvement of (severe) constipation as well as effects on small intestine bacterial overgrowth (SIBO) and motor impairment [211,212,213]. A double-blind, placebo-controlled study to evaluate effects of FMT in PD is currently in progress [214]. In AD, two case reports described a considerable improvement of cognitive function in AD patients with comorbid CDI following FMT treatment [215,216]. Unfortunately, a planned clinical trial has been terminated due to the detection of SARS-CoV-2 in the feces [217], revealing the major limitation of this treatment. Taken together, the existing data on the effect of FMT on PD and AD patients are too limited to support a broader application of FMT in these two diseases [218]. Moreover, the most promising route of administration (nasogastric/-duodenal or rectal) has to be investigated in the future.

### 5.5. Medication

Apart from the well-known effects of antibiotics on the microbiome [219], a multitude of studies showed that other drug classes, including antidiabetics (metformin), psychiatric medication (antidepressants), or proton pump inhibitors influence the gut microbiome [153,220,221]. In turn, the effects of patient medication can be compromised by gut microbiota metabolization as shown for L-dopa in PD [222]. However, further studies are needed to determine whether specific medication can be used to yield microbiome-mediated positive effects on neurodegenerative diseases.

Taken together, the microbiome is an interesting therapeutic target, especially considering the possibility of easily applicable low-risk interventional therapies. However, it has to be taken into account that most of the microbiome studies in AD and PD have been conducted in the clinical phase of the diseases, with the goal of disease modification. Causal treatment, however, should directly target underlying pathomechanisms occuring in the prodromal/preclinical phases of neurodegeneration, which, in terms of microbiome alterations, have yet to be elucidated. So far, the evidence for a medical use of probiotics/prebiotics or FMT is not sufficient, especially considering rare but possible side-effects or adverse reactions of these treatments. However, considering the wide-ranging beneficial effects of a healthy lifestyle, recommendations on diet and exercise, potentially influencing microbiome-driven pathology in PD and AD, should be further explored.

## 6. Summary and Future Research Perspectives

Both PD and AD, the two most common neurodegenerative diseases, have been associated with substantial changes in the microbiome and metabolome composition in comparison to healthy individuals. Nevertheless, the prognostic value of these changes for early disease diagnosis and prognosis, most particularly in the prodromal disease phases, still needs to be determined in large-scale, prospective multi-omics studies. On the other hand, both the microbiome and the metabolome might offer novel therapeutic targets for effective disease treatment. This, however, requires a deep, systematic understanding of the underlying disease pathomechanisms to improve patient outcomes and minimize side-effects. A systems-wide understanding of the GI–brain axis covering not only individual organs and/or biocompartments, but comprising the whole organism, would potentially facilitate these insights. Systems medicine and systems biological modeling approaches of host-microbiome interactions represent key tools in achieving such a systematic understanding. On the sampling side, however, both microbiome and metabolome analyses in PD and AD have been limited to individual organs and mainly standard biospecimens such as feces and blood. The additional multi-omics investigation of other biospecimens such as saliva and CSF might, in particular, offer novel insights into the link between the oral microbiome, the GI–brain axis, and neurodegeneration. Many options to actively change the microbiome and metabolome composition are already available, most importantly dietary interventions and lifestyle changes. However, their downstream effects on neurodegeneration still need to be explored. With respect to the long prodromal phases of PD and AD, a major focus should be set on affordable and low-threshold interventions.

Finally, systematic comparisons of microbiome and metabolome changes across different neurodegenerative diseases both cross-sectionally and longitudinally are still lacking. An extension of these systematic comparisons to other diseases, in which neurodegeneration contributes to disease progression and symptoms, such as multiple sclerosis or Huntington’s disease, might unveil disease-overarching pathomechanisms of neurodegeneration. In fact, profound microbiome and metabolome changes have already been reported in both multiple sclerosis [223,224], which is in addition to the inflammatory pathophysiology characterized by profound neurodegeneration, and Huntington’s disease [225]. Thus, deeply phenotyped multi-cohort studies as well as clinical trials including several distinct neurodegenerative diseases such as PD, AD, multiple sclerosis, and/or rarer neurodegenerative diseases such as Huntington’s disease might pave the way to a deeper mechanistic understanding of neurodegeneration and uncover novel therapeutic strategies to fight this global pandemic.

## Abbreviations

AD	Alzheimer’s disease
BCAA	branched chain amino acid
BMI	body-mass-index
CE-FTMS	capillary electrophoresis Fourier transform mass spectrometry
CSF	cerebrospinal fluid
FT-ICR-MS	Fourier transform ion cyclotron resonance mass spectrometry
GC-MS	gas chromatography mass spectrometry
LC-MS	liquid chromatography mass spectrometry
LC-MS/MS	liquid chromatography tandem mass spectrometry
MCI	mild cognitive impairment
MRI	magnetic resonance imaging
NMR	nuclear magnetic resonance
PD	Parkinson’s disease
PET	positron emission tomography
SCFA	short-chain fatty acid
TMAO	trimethylamine-N-oxide
